# Supplementary material for: The Systems Biology Research Tool: evolvable open-source software
Source: BMC Syst Biol. 2008 Jun 29;2:55. doi: 10.1186/1752-0509-2-55 (PMC2446383; doi:10.1186/1752-0509-2-55)
Supplement: Additional file 1 — SBRT Archive. An archive of the current version of the Systems Biology Research Tool. [file 1752-0509-2-55-S1.zip › sbrt-1.4.0/doc/developers_guide/api/sbrt/shell/io/SbrtFile.html]

SbrtFile


|  |  |  |
| --- | --- | --- |
| **Overview** \| **Package** \| **Class** \| **Use** \| **Tree** \| **Deprecated** \| **Index** \| **Help** | |  |
| **PREV CLASS**   **NEXT CLASS** | **FRAMES**    **NO FRAMES**     **All Classes** |  |
| SUMMARY: NESTED \| FIELD \| CONSTR \| METHOD | DETAIL: FIELD \| CONSTR \| METHOD |  |


---


## sbrt.shell.io Interface SbrtFile

**All Known Subinterfaces:**: FbaOptOutputFile, InputFile<E>, MapInputFile<K,V>, MapOutputFile<K,V>, MapTextFile<K,V>, MapTextInputFile<K,V>, MapTextOutputFile<K,V>, OutputFile<T>

**All Known Implementing Classes:**: AbstractMapInputFile, AbstractMapOutputFile, ColumnOrFbaOptOutputFile, ComiFile, ComoFile, FluxCapInputFile, FluxIntervalInputFile, ProcessInputFile, RomiFile, RomoFile, RowOrFbaOptOutputFile, RxnNamExprSetInputFile, SerFbaOptOutputFile, SerializedInputFile, SerializedMapInputFile, SerializedOutputFile, SmoFile, TextInputFile, TextOutputFile

---

``` public interface SbrtFile ```

This interface is used to represent files.

**Author:**
:   This interface was written and documented by
    Jeremiah Wright while in the Wagner lab.

---

| **Method Summary** | |
| --- | --- |
| `void` | `close()`             Closes this file. |
| `java.lang.String` | `getCanonicalName()`             Returns the canonical name of this file. |
| `java.lang.String` | `getOriginalName()`             Returns the original name given to this file. |
| `boolean` | `isClosed()`             Indicates if this file has already been closed. |

**Method Detail**

### close

```
void close()
```

:   Closes this file. If this file is already closed, then
    invoking this method has no effect.

---


### isClosed

```
boolean isClosed()
```

:   Indicates if this file has already been closed.

    :   **Returns:**: `true` if this file is closed; `false` othewise.

---


### getOriginalName

```
java.lang.String getOriginalName()
```

:   Returns the original name given to this file.

    :   **Returns:**: the original name given to this file.

---


### getCanonicalName

```
java.lang.String getCanonicalName()
```

:   Returns the canonical name of this file.

    :   **Returns:**: the canonical name of this file. **See Also:**: `File.getCanonicalPath()`


---


|  |  |  |
| --- | --- | --- |
| **Overview** \| **Package** \| **Class** \| **Use** \| **Tree** \| **Deprecated** \| **Index** \| **Help** | |  |
| **PREV CLASS**   **NEXT CLASS** | **FRAMES**    **NO FRAMES**     **All Classes** |  |
| SUMMARY: NESTED \| FIELD \| CONSTR \| METHOD | DETAIL: FIELD \| CONSTR \| METHOD |  |


---
